# SUMO Wrestles with Recombination

**DOI:** 10.3390/biom2030350

**Published:** 2012-07-25

**Authors:** Veronika Altmannová, Peter Kolesár, Lumír Krejčí

**Affiliations:** 1 Department of Biology, Masaryk University, Brno 62500, Czech Republic; Emails: 77543@mail.muni.cz (V.A.); 107232@mail.muni.cz (P.K.); 2 National Centre for Biomolecular Research, Masaryk University, Brno 62500, Czech Republic; 3 International Clinical Research Center, Center for Biomolecular and Cellular Engineering, St. Anne’s University Hospital in Brno, Brno 62500, Czech Republic

**Keywords:** SUMO, homologous recombination, double-strand break, meiosis

## Abstract

DNA double-strand breaks (DSBs) comprise one of the most toxic DNA lesions, as the failure to repair a single DSB has detrimental consequences on the cell. Homologous recombination (HR) constitutes an error-free repair pathway for the repair of DSBs. On the other hand, when uncontrolled, HR can lead to genome rearrangements and needs to be tightly regulated. In recent years, several proteins involved in different steps of HR have been shown to undergo modification by small ubiquitin-like modifier (SUMO) peptide and it has been suggested that deficient sumoylation impairs the progression of HR. This review addresses specific effects of sumoylation on the properties of various HR proteins and describes its importance for the homeostasis of DNA repetitive sequences. The article further illustrates the role of sumoylation in meiotic recombination and the interplay between SUMO and other post-translational modifications.

## 1. Introduction

Maintenance of genetic information is essential for genomic integrity, but it is constantly challenged by enormous amounts of DNA damage. DNA double-strand breaks (DSBs) comprise one of the most serious kinds of DNA lesions. If left unrepaired, these could lead to aneuploidy, genetic aberrations or cell death. Defects in DSB repair are linked to many human syndromes, such as neurodegenerative diseases, immunodeficiency and cancer. Two major pathways have evolved for repair of DSBs: non-homologous end joining (NHEJ) and homologous recombination (HR). The error-prone NHEJ is used throughout the cell cycle and is most prominent in G1 phase of the cell cycle. On the other hand, the error-free HR generally uses sister chromatid for repair and is therefore dominant in the S and G2 phases [1,2,3]. The pathway choice between the DSB repair mechanisms also varies among species. This could reflect different expression of various recombination proteins, presence of factors that suppress or promote individual pathway as well as regulation on the level of post-translational modifications [1,2,3,4,5,6,7]. Increasing evidence indicates that not only phosphorylation, but also sumoylation is possibly a key regulatory component. Here, we review the role of sumoylation during DSB repair while focusing especially on the HR pathway. For clarity’s sake, we predominantly summarize findings from yeast, as this model system provides most of the pioneering studies, but we will also integrate these findings with data from other organisms. 

### 1.1. Double-Strand Break Repair

**Figure 1:**
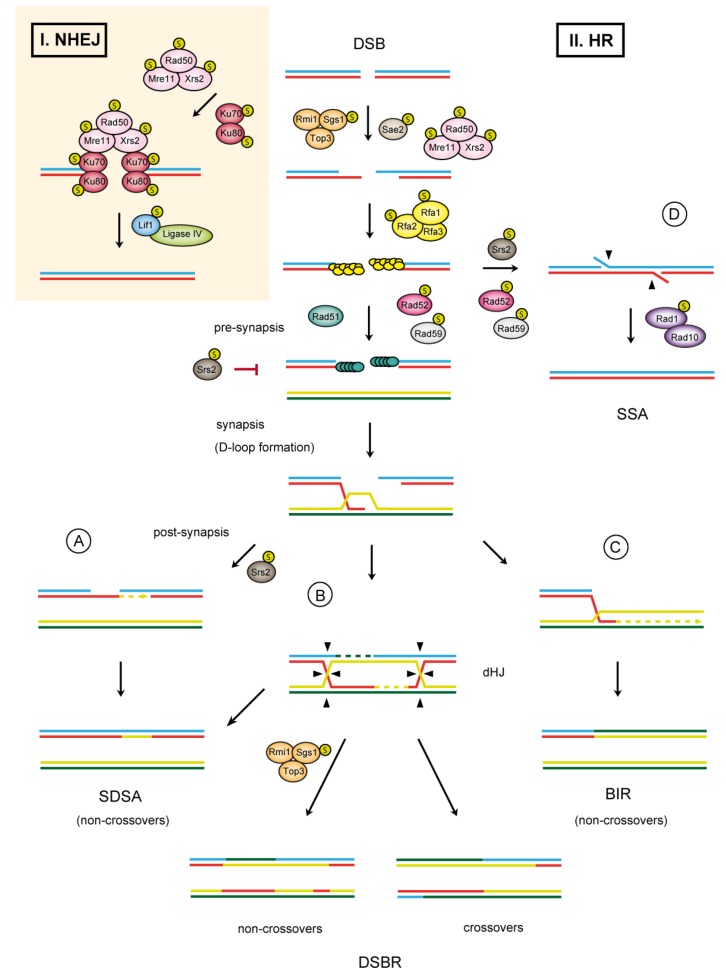
The double-strand break repair pathways in *S. cerevisiae*. After DNA damage, DSBs can be either resected to generate 3’ ssDNA tails and directly ligated by non-homologous end joining (NHEJ) (I) or processed by homologous recombination (HR) (II). In HR, resection of a DSB is followed by formation of a Rad51 presynaptic filament invading into the homologous strand to form a D-loop structure. The invading strand is then extended by DNA synthesis. The resulting extended D-loop could then be processed by one of three alternative mechanisms: synthesis-dependent strand annealing (SDSA) (**A**); double-strand break repair (DSBR) (**B**); or break-induced replication (BIR) (**C**); Proteins involved in DSB repair that undergo sumoylation are depicted. An alternative pathway–single strand annealing (SSA)–can be used for DSBs occurring between repeated DNA sequences (**D**).

The two pathways for DSB repair differ in their mechanisms and enzymatic requirements. The repair of DSBs via NHEJ is characterized by binding of the Ku70/80 heterodimer to the broken ends followed by recruitment of the Mre11-Rad50-Xrs2 (MRX) complex (Figure 1). The main function of the Ku70/80 complex is to protect DNA ends against nucleolytic degradation and to recruit additional NHEJ proteins. Meanwhile, MRX complex bridges the ends and prevents their separation. In some cases, the ends require removal of damaged nucleotides to allow conjugation by NHEJ-specific DNA ligase IV and its associated factor, Lif1 (reviewed in [8]; Figure 1). 

In the second DSB repair pathway, homologous recombination (Figure 1), the broken ends need to be nucleolytically processed to produce 3’ single-stranded DNA (ssDNA) overhangs. During HR, MRX complex binding to the broken ends catalyzes removal of short oligonucleotides from the 5’ ends in collaboration with Sae2 nuclease. Two alternative pathways then extensively process the short 3’ overhangs. One is characterized by an action of Exo1 (5’–3’ exonuclease), while the other is dependent on the activities of Dna2 endonuclease and the Sgs1-Top3-Rmi1 complex [9]. During resection, the resulting ssDNA strand is rapidly bound by replication protein A (RPA), which not only promotes end resection but also prevents formation of secondary structures. In addition, RPA creates a barrier for the binding of Rad51 recombinase, thus prohibiting the formation of Rad51 presynaptic filament. To overcome this inhibitory effect, the action of recombination mediators, in particular Rad52 and the Rad55–57 complex, is required [10]. These assemble Rad51 on the RPA-coated ssDNA and promote the formation of Rad51 filament. Upon Rad51 filament formation, HR continues by searching for a homologous sequence, followed by DNA-strand invasion, and results in a D-loop formation. These reactions are catalyzed by Rad54 protein [11]. 

The invading strand of the D-loop is at this point extended by a polymerase, which is followed by resolution in one of the three possible sub-pathways. The first pathway, designated double-strand break repair (DSBR, [12]), proceeds by capturing the second DSB end to the extended D-loop and formation of double Holliday junctions (dHJ) (Figure 1B). dHJs are then resolved into either crossover or non-crossover products characteristic of meiotic recombination (recombination in meiosis will be discussed later). The second pathway, synthesis-dependent strand annealing (SDSA, [13]), is characterized by displacement of the extended strand from the D-loop and its consequent annealing with the complementary strand of the other resected DSB end (Figure 1A). This pathway eliminates formation of crossovers and is therefore typical for the repair of DSBs during mitosis. In the third alternative pathway, the D-loop assembles into a full-fledged replication fork in a process called break-induced replication (BIR) (Figure 1C).This mechanism can lead to a loss of heterozygosity and is also often used to repair broken or shortened telomeres [14]. For further details about HR, see additional review articles [15,16,17,18].

DSBs can alternatively be repaired by a mechanism known as single-strand annealing (SSA). SSA is used for DSB repair if directly repeated DNA sequences are present (Figure 1D). After the resection of DSBs, the generated single-stranded DNA overhangs can anneal to the complementary DNA strand with the help of Rad52 and Rad59 proteins. The 3’ non-homologous tails, produced during annealing as an intermediate, are removed by the Rad1-Rad10 endonuclease followed by gap filling and ligation (reviewed in [17]).

### 1.2. SUMO

Sumoylation is a post-translational modification characterized by an attachment of SUMO (small ubiquitin-like modifier) peptide to target proteins. SUMO, known as Smt3 in *S. cerevisiae*, is an 11 kDa protein that modifies many proteins participating in diverse cellular processes, including DNA repair, replication, basic metabolism, gene transcription, ion and protein transport, and others [19,20,21,22] The conjugation of SUMO to a target protein involves a 3-step mechanism analogous to ubiquitylation (reviewed in [19,20,21,22]). It is initiated by an ATP-dependent activation of the SUMO protein by SUMO-activating enzyme (E1), a heterodimer consisting of Aos1 and Uba2. SUMO is then transferred to SUMO-conjugating enzyme (E2), Ubc9, which catalyzes the conjugation of SUMO to a target protein. To accomplish efficient and specific sumoylation of the substrate, however, the presence of SUMO ligases (E3) is usually necessary [20,23]. To date, four SUMO E3 ligases have been identified in budding yeast: Siz1, Siz2, Mms21, and meiosis specific Zip3 [20,23,24,25,26]. Attachment of SUMO can block interactions occurring at or near the attachment site, or, more often, it provides a binding surface for new protein interactions or stimulates the existing ones. In the latter case, the binding protein contains a SUMO-specific binding site, known as SIM (SUMO-interacting motif). The SIM has been shown to play essential role in multi-step enzymatic processes, and affect the assembly and disassembly of dimeric and multimeric protein complexes (reviewed in [27]). The crucial component of SIM is a hydrophobic amino acid core, which provides an interface for non-covalent interaction with SUMO. However, also position of acidic residues juxtaposed to SIM can further stimulate SIM-SUMO interaction [27,28,29,30,31]. Alternatively, negative charge in the SIM can be introduced by phosphorylation of serine or threonine residues and can additionally lead to increased selectivity of the protein interaction [30,32,33]. At functional level, SUMO has been shown to change protein interaction, localization, stability or activity [19,20,21]. Importantly, sumoylation is a reversible process and SUMO can be rapidly deconjugated from the target protein by the action of SUMO-specific proteases (Ulp1 and Ulp2 in yeast), which makes sumoylation ideal for regulatory purposes [19].

### 2. SUMO in Recombination

As mentioned above, SUMO modification has been implicated as a possible key player in the regulation of DSB repair. Mutations or deletions of the components of SUMO machinery lead to severe defects, including recombination abnormalities, thus implicating sumoylation as a potential regulator of recombinational repair [25,34,35,36,37]. Ubc9 mutant cells as well as cells carrying a SUMO ligase-deficient allele of *MMS21* exhibit increased sensitivity to DNA-damaging agents [25,35]. Moreover, during replication of damaged template both mutants accumulate cruciform structures in a Rad51-dependent manner [34]. In addition, a strong hyper-recombination phenotype has been observed in a mutant of SUMO protease Ulp1. This mutant has also been shown to be synthetically lethal with mutations in genes involved in HR (such as *srs2, rad51, rad52, rad54, rad55, rad50*, and *mre11*) [37]. 

Recently, a breakthrough study from the Zhao laboratory has revealed a comprehensive role of sumoylation in maintaining genome stability [38]. Using a biochemical screen in yeast, they identified a large group of proteins participating in DNA repair and undergoing sumoylation, mainly in response to DNA damage. This revelation greatly broadens the potential roles of sumoylation in genome maintenance. Additional discussion on this work will be described in another review article by Zhao *et al*. in the next special issue focusing on “DNA damage response”.

### 2.1. SUMO in HR

In HR, the spectrum of SUMO-modified proteins includes all steps indicating SUMO’s substantial role in HR regulation (see Table 1). However, the exact role of sumoylation in HR regulation is often elusive, due to the problems in identification of modified sites and their possible redundancy. Identification of the downstream SUMO-interacting partners, analysis of sumo-deficient alleles as well as permanent sumo-fusion of target proteins that can only partially mimic the effect of sumoylation further hinder the task. 

**Table 1:** Sumoylated proteins involved in DSB repair.

Pathway	Yeast	Human	Function	Effect of sumoylation	Reference
**NHEJ**	Ku70	KU70	subunit of Ku complex, protection of DNA ends, recruitment of other NHEJ factors	unknown	[25,64]
	Ku80	KU80	subunit of Ku complex, protection of DNA ends, recruitment of other NHEJ factors	unknown	[38,65]
	Lif1	XRCC4	DNA ligation	intracellular localization (human)	[38,66]
**HR**	Mre11 ^1^	MRE11	subunit of MRX complex (DSB resection)	unknown	[38]
	Rad50 ^1^	RAD50	subunit of MRX complex (DSB resection)	unknown	[38]
	Xrs2 ^1^	NBS1	subunit of MRX complex (DSB resection)	unknown	[38]
	Sae2	CtIP	DSB resection	unknown	[38]
	Rad52 ^2^	RAD52	recombination mediator	inhibition of biochemical activities, intranuclear localization, protein stability (yeast)	[42,43]
				subcellular localization (human)	
	RPA ^2^	RPA	binding resected DNA tails	recruitment of RAD51 to initiate HR (human)	[48,67]
	Rad59 ^2^		stabilization of Rad51 filament, ssDNA annealing	unknown	[67]
	Sgs1	BLM	RecQ-like helicase, resolution of dHJ	Sgs1 sumoylation stimulates recombination at telomeres	[34,68]
				BLM sumoylation promotes Rad51-dependent recombination	
		WRN	RecQ-like helicase, resolution of dHJ	WRN sumoylation affects its nuclear localization	[69,70]
	Srs2		helicase, disruption of Rad51 filament, promoting SDSA	unscheduled sumoylation in	[54]
				non-phosphorylatable Srs2 causes recombinational repair defects	
**SSA**	Rad1	XPF	subunit of Rad1–Rad10 complex (nuclease activity)	unknown	[38]

^1^ also involved in NHEJ and SSA; ^2^ also involved in SSA.

Nevertheless, the available data of selected examples described below show the diversity of the effects of SUMO modification, including its ability to regulate the intracellular localization, stability, and conformation of target protein as well as their interactions or biochemical activities. Future studies will be needed to uncover the molecular mechanism and biological function of sumoylation in HR.

#### 2.1.1. DNA End Resection

The initiation of the end resection turns the DSB repair into HR pathway. The MRX and Sae2 represent the key components of end processing machinery. The role of sumoylation in this step is clearly indicated by DNA damage induced SUMO targeting of these proteins [38]. Furthermore, defective sumoylation results in an impaired DNA end resection, suggesting that recombination is facilitated by sumoylation [38]. This further supported by the fact that the deletion of Mre11 (leading to disruption of the MRX complex) causes decreased sumoylation of several downstream proteins participating in presynaptic filament formation, such as Rad52, Rad59, Rfa1 and Rfa2 [38]. Perhaps the extent of ssDNA generated through the end processing is being monitored and correlated by DNA damage-induced sumoylation machinery. This is an interesting reminiscence of DNA-damage checkpoint signalling in human that is sensed by ATR-ATRIP complex via extent of ssDNA bound by RPA [39,40]. Further discussion on the relationship between the two will be covered in the above-mentioned review in next issue.

#### 2.1.2. Presynaptic Filament Formation

Sumoylation strikes at the heart of HR by modifying the crucial recombination mediator Rad52. SUMO-modified Rad52 has been found in *S. cerevisiae*, *S. pombe* and human cells indicating a conservation of this process [41,42]. However, SUMO conjugation sites (K10, 11, and 220) identified in *S. cerevisiae* Rad52 are located outside the highly conserved domain and sumoylation patterns are obviously different in yeast and human Rad52 leading to a hypothesis that sumoylation may have various regulatory roles in yeast and mammalian cells [42,43]. Studies from budding yeast have shown that sumoylated Rad52 is DNA damage-induced and occurs in mitotic as well as meiotic cells [42]. 

Nonsumoylatable Rad52 exhibits no significant hypersensitivity to MMS and is also not defective in spore viability and sporulation, indicating that SUMO modification maintains Rad52 function [42]. Correspondingly, impaired sumoylation of Rad52 does not significantly affect major mitotic and meiotic recombination frequencies but rather influences the choice and efficiency of the recombination pathway with slight shift towards SSA in sumoylation defective *rad52 mutant* [44]. This might reflect the defects in biochemical properties of sumoylated Rad52 such as decreased DNA binding, annealing activity and corresponding shorter duration of *rad52* sumo-deficient foci [44]. In addition, ssDNA stimulates Rad52 sumoylation and this is not blocked when coated by RPA [44]. This is in good correlation with reduced Rad52 sumoylation in mutants of MRX complex that fail to generate ssDNA due to block of DSB end processing [38,42]. On the other hand ssDNA coated by Rad51 protein is not anymore capable of stimulating Rad52 sumoylation indicating that Rad52 sumoylation proceeds prior to Rad51 filament formation [44]. This hypothesis is further supported by the fact that deletion of *RAD51* leads to accumulation of sumoylated Rad52, while deletion of factors participating in subsequent steps of HR (such as Sgs1, Srs2, Rad55, Rad54, Rad59) or replacing Rad51 with an ATPase defective Rad51-K191R mutant suppresses this effect [45]. This evokes an attractive possibility that Rad51-dependent reactions require sumoylated Rad52. Further, accumulation of Rad51-intermediates results in desumoylation of Rad52 leading to its increased proteasomal degradation, a phenotype observed for sumoylation-defective Rad52. This behaviour was even more pronounced in double mutants of Srs2, Sgs1, or Rrm3 helicases, which are known to accumulate recombination intermediates and loss of Rad52 function rescues the cell growth [42]. Moreover, sumoylated Rad52 has been found as an *in vitro* substrate for Slx5–Slx8 complex, which is a member of SUMO-Targeted Ubiquitin Ligase (STUbL) family of proteins [46]. Slx5 and Slx8 are both RING finger proteins containing multiple SUMO-interacting motifs for binding to conjugated SUMO on a target protein. Such interaction can serve as a signal for Slx5-Slx8-mediated ubiquitylation that could potentially lead to ubiquitin-dependent degradation [47]. However, neither *slx5* nor *slx8* cells display slower degradation of SUMO-fused Rad52 indicating a possible different function of Slx5–Slx8-mediated ubiquitylation for sumoylated Rad52 [46]. 

As mentioned above, the single-stranded DNA-binding protein RPA is also a target for sumoylation and SUMO has also been observed to modify its mammalian homolog [48]. While the role of RPA sumoylation in yeast has not yet been addressed, studies of human RPA support the pro-recombination role of sumoylation as SUMO-modified RPA70 initiates Rad51-dependent HR. After treatment with the replication stress inducer camptothecin, RPA70 dissociates from SUMO-specific protease SENP6 and is modified by SUMO-2/3 thus increasing its association with RAD51. This enhancement could be due to interaction of RAD51 with SUMO. Sumoylated RPA70 then facilitates formation of RAD51 foci and promotes HR [48]. Interestingly, the observation that RAD51 interacts with SUMO and also with UBC9 provokes an idea about potential regulation of RAD51 filament formation by sumoylation in humans [49,50]. Nevertheless, sumoylation of Rad51 has not yet been observed either in yeast or in mammals.

The effect of recombination mediators can be counteracted by the helicase Srs2, which potently dismantles Rad51 filaments to prevent inappropriate recombination [51,52] and Srs2 is also sumoylated in response to DNA damage [53,54]. Though the function of Srs2 modification is unclear, unscheduled sumoylation has been found to impair SDSA in non‑phosphorylatable Srs2 mutant [54]. Moreover, sumoylation of Srs2 modifies its affinity towards SUMO-PCNA and might be involved in regulation of the diverse roles of Srs2 (the interplay between Srs2 and SUMO-PCNA will be discussed later) [53]. 

#### 2.1.3. Synaptic Phase

During synapsis, Rad51 presynaptic filament is stabilized by Rad54 protein (a member of the Snf2/Swi2 family of DNA-dependent ATPases), which further stimulates DNA-strand invasion and D-loop formation [55,56]. Even though sumoylation of Rad54 has not been observed, another member of the Snf2/Swi2 family, Uls1, has been proposed to be a SUMO-targeted ubiquitin ligase (STUbL) [57]. Uls1 is reported to bind both SUMO and the ubiquitin-conjugating enzyme Ubc4 and is required to ubiquitylate SUMO conjugates [57]. Nevertheless, the biochemical evidence about its SUMO-dependent ubiquitylation activity is still missing. Importantly, together with other translocases-Rad54 and Rdh54, Uls1 is important for the removal of Rad51 recombinase from chromatin [58]. Recently, Uls1 has been also implicated in replication stress response, and especially in cells lacking Rad52 mediator proteins or Mus81/Mms4 nuclease [59]. However, additional studies will be required to clarify the function of Uls1 as a STUbL ligase potentially targeting HR proteins. 

#### 2.1.4. Post-Synaptic Phase

SUMO also modifies proteins participating in the post-synaptic phase of HR, including members of the RecQ helicase family, which are involved in resolution of recombination intermediates. Sumoylation of Sgs1 helicase is stimulated by DSB formation induced by ionizing radiation or chemicals [60]. The observation that mutations in Ubc9, Mms21 and Sgs1 results in similar phenotypic outcome suggests that Sgs1 sumoylation is important for resolution of the X-shaped structures formed during DNA replication [34]. It is noteworthy that sumoylation of Sgs1 at K621 was found to be dispensable for homologous recombination but functionally important for telomere–telomere recombination [60]. Studies from other organisms have shown that SUMO modification is a conserved mechanism among RecQ helicases. Sumoylation of Rqh1, a Sgs1 orthologue in *Schizosaccharomyces pombe*, controls its activity at telomeres [61]. Further, SUMO also regulates the pro- and anti-recombinogenic roles of the human Sgs1 ortholog BLM (deficient in Bloom syndrome). The cells expressing SUMO-deficient BLM mutant are defective in HR and display a defect in Rad51 localization to stalled replication forks [62].

### 2.2. SUMO in NHEJ

The role of SUMO in DSB repair is not restricted to HR, as proteins involved in NHEJ undergo sumoylation as well. Interestingly, a SIM-containing peptide has been found to inhibit NHEJ in humans cell lines, though, the underlying mechanism remains unknown [63]. Ku70 protein, which forms a heterodimer with Ku80, has been shown to be sumoylated in both yeast and humans and can also interact with the SIM-containing peptide after radiation [25,63]. Since the main role of Ku70 is to recognize and protect DSBs as well as load other NHEJ factors, it is possible that SUMO-SIM mediated interaction can either affect the dynamics of the recruitment of additional NHEJ proteins or regulate the removal of the Ku70/80 heterodimer from DNA ends. Also sumoylation of Lif1 and MRX complex was recently observed, but the biological function is not known [38]. Interestingly, the sumoylation of NHEJ factors (Ku70, Ku80 and Lif1) was not influenced by deletion of Mre11 in contrast to proteins involved in recombinational repair, suggesting that resection contributes to the HR proteins sumoylation induction [38]. Therefore it might be intriguing to speculate if sumoylation might play important role in pathway choice between NHEJ–HR, or consequent amplification of the decision signal. 

## 3. The Interplay of HR and SUMO at the Repetitive Sequences

Though HR plays a major role in the repair of the DNA containing repetitive sequences, it has to be tightly regulated, as the presence of multiple homologous sequences can lead to unequal sister chromatid exchange and subsequent loss of genetic information. SUMOylation may represent one of the control mechanisms at the repetitive sequences and we will illustrate this on examples of the ribosomal (rDNA) and telomeric DNA.

### 3.1. SUMO and the rDNA

The rDNA is localized in the nucleolus and in S. *cerevisiae* is formed by 150 tandem repeats encoding the 35S and 5S ribosomal RNAs. Sumoylation plays an essential role in regulation of rDNA recombination as the loss of sumoylation severely impairs rDNA stability [71,72]. The important role of SUMO is further suggested by the striking localization of sumoylated proteins in the nucleolus, when SUMO deconjugation is blocked [73]. One main target of SUMO regulation of HR in nucleolus is the recombination mediator Rad52. In wild type cells, sumoylated Rad52 is excluded from the nucleolus and the DSB is repaired in the nucleoplasm. In contrast, SUMO-deficient mutant of Rad52 forms foci within the nucleolus resulting in hyperrecombination at the rDNA locus and rDNA marker loss [72]. Rad52 sumoylation therefore seems to specifically inhibit formation of Rad52 foci in the nucleolus in order to preserve rDNA integrity. Similar phenotype have been also observed in the Smc5–Smc6 mutant cells [72]. Smc5-Smc6 (structural maintenance of chromosomes) heterodimer forms a core of a multimeric complex containing also six non-Smc elements (Nse1–Nse6) [74]. The complex regulates sister chromatid cohesion, HR, chromatin structure and its dynamics, however, many aspects of its function still remain unclear [74,75]. Smc5–Smc6 is highly enriched at the rDNA and other repetitive sequences and is required for their proper segregation. The complex associates with E3 SUMO ligase Mms21 (Nse2), which activity is required for the integrity of repetitive sequences [25,76]. However, Mms21 in the nucleolus probably targets other factors than Rad52, as Rad52 sumoylation is independent of the Smc5–Smc6 complex and they rather act synergistically in limiting recombination at rDNA [72]. Another protein complex that is enriched in the nucleolus and is involved in the rDNA recombination regulation is the Slx5–Slx8 SUMO-targeted ubiquitin ligase. The *slx8Δ* cells exhibit increased rDNA recombination and nucleolar Rad52 foci formation [67]. The *slx8Δ* cells also accumulate Smt3 foci in the nucleolus, suggesting the role of Slx5–Slx8 in proteasomal degradation of sumoylated nucleolar proteins [71]. Altogether these findings demonstrate SUMO’s central role in controlling HR at the rDNA locus. However, further studies are required to determine specificity of the DNA-damage response in nucleolus, other nucleolus-specific SUMO targets, dynamics and molecular mechanism of protein re-localization.

### 3.2. SUMO and the Telomeres

Telomeres-the structures located at the ends of chromosomes, are vital for the stability and complete replication of the genome. In *S. cerevisiae* they consist of 300 base pairs of telomeric repeats terminated by short 3’ single-stranded overhangs. Their resemblance to DSBs necessitates specific set of proteins that protects telomeres from recognition as DSBs and against exonucleolytic cleavage [77]. 

SUMO is an important regulator of the telomeres and many telomeric proteins undergo sumoylation [78]. Sumoylation limits telomere length both in budding and fission yeast [25,78,79,80,81]. A main target of sumoylation is the Cdc13 protein, an important telomerase regulator. Siz-dependent sumoylation of Cdc13 strengthens its interaction with the telomerase inhibitor Stn1 and thus suppresses telomerase function [78]. Siz2 was also shown to sumoylate Ku70/80 and Sir4 proteins and thus stimulate anchoring of telomeres to the nuclear envelope [82]. The observations that *Siz2Δ* mutant displays telomerase–dependent telomere extension and elongating telomeres shift away from the nuclear envelope, led to the hypothesis that sumoylation represses telomerase by tethering telomeres to the nuclear periphery, whereas their release is connected to telomere elongation [82]. The importance of sumoylation in directing telomeres to nuclear envelope is also supported by impaired telomere clustering in sumoylation deficient Mms21 cells [25]. Though the mechanism of SUMO-dependent telomere anchoring to the nuclear envelope remains elusive, it possibly involves multiple SUMO–SIM interactions, as both structures are associated with profound sumoylation. The Smc5–Smc6 complex is enriched at the telomeres in the budding and fission yeast [83,84,85]. The whole complex as well as Mms21 activity are particularly important for telomere maintenance in telomerase deficient cells, as their absence cause accumulation of HR intermediates at telomeres, aberrant recombination between sister telomeres and growth termination [86,87]. 

Contradictory to SUMO’s role in telomere length restriction, SUMO was also found to play an important role in telomere length increase in the telomerase-deficient cells. Though in most cells lacking telomerase the telomere length gradually decreases and finally leads to cell cycle arrest [88], some cells are able to maintain the telomere length by employing recombination-mediated pathways, which are in humans referred to as alternative lengthening of telomeres (ALT) [89]. Sumoylation of Sgs1 was shown to specifically promote telomeric recombination in telomerase-deficient cells [60]. Similarly, sumoylation of Rqh1, the RecQ homologue in *S. pombe*, stimulates ALT-like recombination events in the *taz1Δ* and *taz1Δ trt1Δ* cells [61]. Though the mechanism by which sumoylation of Sgs1 and Rqh1 increases the telomeric recombination is unclear, it was suggested that sumoylation mediates localization of the RecQ helicases to telomeres where they facilitate restart of collapsed replication forks that subsequently lead to telomere-telomere recombination [60,61]. Whether sumoylation of human RecQ homologues has a similar importance in the telomeric recombination remains to be determined. However, conserved sumoylation and the role in telomere maintenance among RecQ helicases indicate their roles may be maintained [68,69,90].

The situation observed in the telomerase-deficient yeasts resembles the one occurring in human ALT cancer cell, and importantly in both sumoylation plays a central role in the promoting of telomeric recombination. Similarly to the absence of telomerase in the *tlc1Δ* yeasts, telomerase is downregulated in most human cells. However, the cancer cells are able to elongate their telomeres and achieve unlimited replicative potential either by upregulating telomerase transcription (85% of cancers), or by the ALT mechanism using recombination between telomeres (15% of cancers) [89,91,92]. The telomeres of the ALT cells usually associate with PML (promyelocytic leukemia) nuclear bodies, which are in this context referred to as ALT-associated PML bodies (APBs) [93]. SUMO plays a central role in the PML bodies as sumoylation and subsequent noncovalent SUMO–SIM interaction between PML subunits is absolutely necessary for PML body formation [94,95]. The association of telomeres and APBs is thought to facilitate recombination between telomeric repetitive sequences, as APBs are known to contain various HR proteins and artificially created APBs cause telomere elongation by a DNA repair mechanism [96,97]. The Smc5–Smc6 complex is also localized in APBs and is required for the telomere–PML colocalization [98]. The necessity of Mms21-dependent sumoylation of telomere-binding proteins for APB formation [98] suggests that the telomere–PML interaction may be stimulated by multiple noncovalent SUMO–SIM interactions, as is the case other PML-interacting partners [68,99,100]. The observation that Smc5–Smc6 depletion inhibits HR at telomeres and their elongation further supports its major role in ALT cells [98]. Moreover, the Smc5–Smc6 complex and Mms21 activity is also important for de-novo formation of PML bodies on telomeric DNA [96,101]. 

The above-mentioned roles of sumoylation in the telomere length restriction and elongation nicely illustrate how sumoylation of the same substrates occurring in different cellular conditions can lead to completely different outcomes. 

## 4. Meiotic Recombination

Recombination during meiosis is a key event that mediates the pairing of homologous maternal and paternal DNA chromosomes, thus ensuring proper exchange of genetic information. Meiotic recombination is characterized by the DSBR pathway that results in generation of crossovers providing a connection between homologues (chiasmata) and facilitating their accurate segregation. In contrast to mitotic recombination, DSBs in meiosis are programmed and endogenously generated by Spo11. After resection, the 3’ ssDNA tails assemble together with Rad51 or meiosis-specific recombinase Dmc1 into nucleoprotein filaments that catalyze a strand-exchange reaction between homologous sequences. While no direct evidence indicates sumoylation of scDmc1, the homologue of Dmc1 in basidiomycete *Coprinus cinereus* (CcLim15) has been shown to interact with Ubc9 and to be sumoylated both *in vitro* and *in vivo* [102]. Another link between SUMO and meiotic recombination is represented by Ecm11 protein. Ecm11 is required for normal DNA synthesis and meiotic recombination, interacts with SUMO and Siz2 ligase, and can also be sumoylated *in vivo* [103,104]. Correspondingly, nonsumoylatable *ecm11* mutant exhibits severe sporulation defects corresponding to the phenotype of the *ecm11Δ* mutant [104]. However the effect on molecular or biochemical activities remains to be determined. The SUMO modification of HR factors during meiosis is not well studied and awaits further characterization. Nevertheless, evidence already exists to suggest its important role for protein stability and function during meiotic DSB repair. 

**Figure 2:**
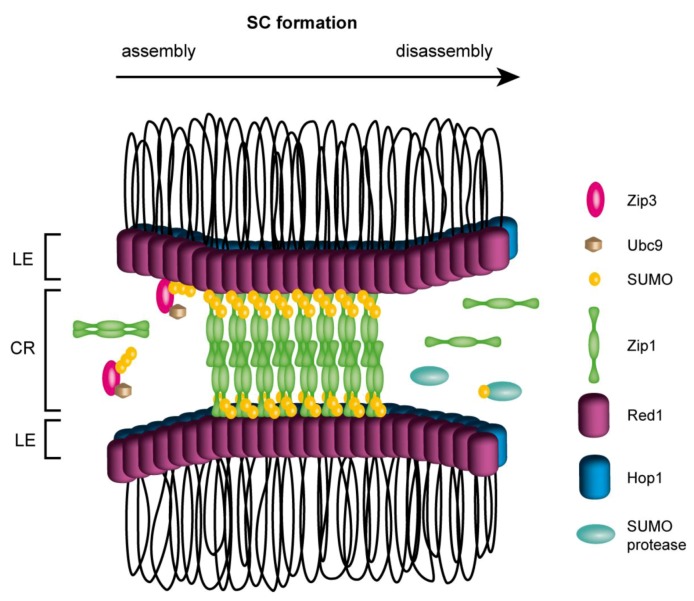
The formation of synaptonemal complex (SC). During SC assembly Zip3 recruits Ubc9 and SUMO to the synapsis sites thus facilitating formation of SUMO chains and conjugation of SUMO to other proteins (such as Red1). Zip1 dimers polymerize along the lateral elements (LE) where they can bind to Red1 and SUMO chains leading to generation of central region (CR). SC disassembly could proceed by dissociation of SUMO conjugates by the action of SUMO proteases or other counteracting mechanism. For clarity, Red1 and Hop1 along LEs are illustrated, even though the exact distribution of Hop1, Red1 and sumoylated Red1 is unknown.

Meiotic recombination proceeds in coordination with the assembly of proteinaceous structures between homologous chromosomes known as synaptonemal complexes (SCs). These structures are formed by two lateral elements (or axes) and a central region which “zips” the axes together. The formation of SCs, which is essential for crossing over, is dependent on initiation of recombination, thus suggesting close connection between recombination and SCs. How the assembly and disassembly of SC is regulated remains unclear (reviewed in [105,106]). Recent observations indicate SUMO as a central player in formation of the SC complex (Figure 2). For example, mutation in Ubc9 leads to delay in synapsis and a major component of the SC central region, the Zip1 protein, has been found to co-localize with SUMO along the synapsed chromosomes [26,107]. Zip1 can also interact with SUMO chains or SUMO-conjugated proteins through the SIM located at its C-terminus [26], suggesting that these interactions may mediate SC formation. This hypothesis is further supported by interaction between Zip1 and the axial element protein Red1. Red1 has been found to interact not only with Zip1 but also with SUMO chains, Ubc9, and SUMO protease Ulp2. These interactions are mediated via the C-terminus of Red1, harboring two SIM motifs [108,109]. The interaction between SUMO chains and Red1 is important for initiating the SC assembly, as it facilitates the Zip1 and Zip3 recruitment [109]. Moreover, the interaction is also essential to promote Tel1- and Mec1-dependent Hop1 phosphorylation, an important step in the cascade promoting interhomologue recombination and ensuring normal meiotic progression [109]. Indeed Red1 can also be covalently modified by SUMO during meiosis [26,108] and its sumoylation seems to be critical for efficient Red1-Zip1 interaction, as interaction Zip1 with SUMO-defective *red1-KR* mutant is significantly decreased. Furthermore *red1-KR* exhibits a substantial delay in SC formation resulting in reduced spore viability. This suggests that SUMO-promoted Red1-Zip1 interaction is important for timely SC formation [108]. 

Eichinger *et al*. have further shown that the level of Red1 sumoylation is impaired in *Δzip3* mutant, thus indicating that Zip3 might be directly linked to Red1’s sumoylation process [108]. It has been shown that Zip3 can function as a SUMO [26] or ubiquitin ligase [110]. As *zip3* mutant accumulate high molecular weight SUMO conjugates similar to *slx5* and *slx8* mutant, it raises an intriguing possibility that Zip3 may also serve as a STUbL [26,46,47,57]. This leads to an interesting hypothesis that coordinated desumoylation can drive disassembly of SC to ensure proper segregation of chromosomes.

Several studies indicate that the role of SUMO in meiosis is conserved among eukaryotes. Similarly to budding yeast Smt3, the SUMO homologue in *S. pombe* (Pmt3) has been found to co-localize along linear elements (LinEs), structures resembling the axial elements of SC [111]. Mutation of the SUMO ligase Pli1 was shown to cause reduced genetic recombination and abnormal LinE formation, thus implicating sumoylation in the regulation of meiotic recombination in *S. pombe* [111].

Further, the putative human functional Red1 homologue SYCP3 has been shown to be modified by SUMO2 [108]. A recent study from mammalian cells also identified SCP1 and SCP2 proteins (SC components) to be conjugated to SUMO1 in human testis [112]. Interestingly, SUMO1 and SUMO-2/3 were found to localize to meiotic chromosomes, but with the distinct patterns of their localization, indicating their separate functions in the cell [112]. Taken together, the aforementioned data implies sumoylation to be a potential key player not only in mitotic and meiotic recombination but also in successful progression and completion of meiosis. 

## 5. Interplay of Post-Translational Modifications

**Figure 3:**
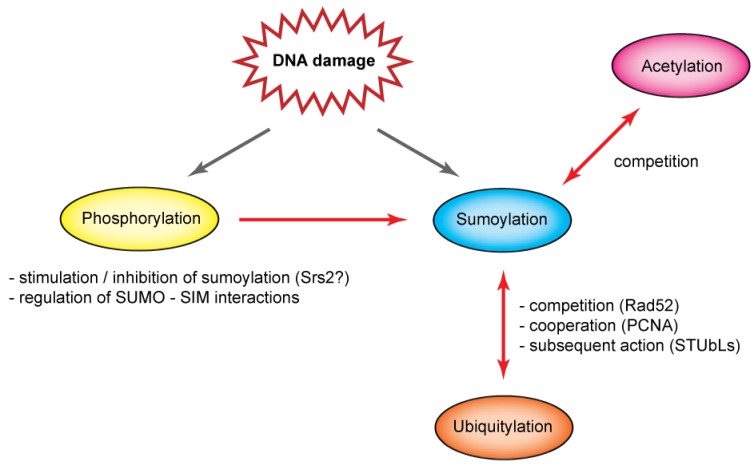
The interplay between sumoylation and other post-translational modifications. Sumoylation does not exist alone but is often influenced by and itself affects other PTMs. Phosphorylation can influence sumoylation both in positive as well as negative manner and also regulate interaction between SUMO and SIM motif in various proteins. Furthermore, acetylation can compete with sumoylation for the same lysine residue, similarly to ubiquitylation that was also reported to cooperate or lead to subsequent reaction with sumoylation. See text for more details.

SUMO is just one component in the intricate network of post-translational protein modifications (PTMs) (Figure 3). Through the years, many examples of various types of interplay between SUMO and other PTMs have been suggested, and these can occur at different levels. Individual pathways can either interact by modifying the same substrate or the modification can target an enzyme belonging to another PTM pathway and thus regulate that PTM pathway’s activity. Here, however, we will only outline the PTM interplay at substrate level. Where this cannot be currently directly illustrated on proteins belonging to the HR pathway, examples from other pathways will be also used. 

First, phosphorylation of multiple proteins can stimulate their sumoylation (Figure 3) [113]. In this case, a kinase usually targets a serine residue situated at a specific phosphorylation-dependent sumoylation motif (PDSM) and leads to stimulation of the Ubc9 binding by creating negative charge [114]. In other cases, phosphorylation inhibits protein sumoylation. For example, it may counteract unscheduled sumoylation, as in the case of Srs2 [54,113]. In addition, phosphorylation has been reported also to regulate SUMO–SIM interactions, a feature seen in the SUMO ligase PIAS1 or the Daxx protein [32,33]. While phosphorylation is known to regulate various metabolic processes it remains to be determined whether it plays wider role in the regulation of sumoylation during recombination. Second, PTM by acetylation often targets the same lysine residues as does sumoylation. This creates competition for the substrate, as illustrated on the histone modifications (Figure 3) [113,115]. Finally, ubiquitin, SUMO’s most famous cousin, can also modify the same lysine residues (Figure 3). It has been suggested, for example, that SUMO could protect the substrate from ubiquitylation and proteasomal degradation, as reported for Rad52 protein [42]. It seems, however, that in most cases SUMO and ubiquitin do not compete for the substrate; they rather act together to take advantage of individual regulations depending on the actual needs of the cell [116]. Sumoylation and ubiquitylation can even act sequentially as seen in such STUbL ligases as Slx5–Slx8 complex and possibly Uls1, which selectively ubiquitylate sumoylated proteins and have been implicated in HR [57].

The complex regulation of DNA repair by PTMs can be ideally illustrated on the proliferating cell nuclear antigen (PCNA). PCNA is a ring-shaped homotrimeric protein that is loaded on DNA to constitute a sliding clamp of DNA polymerases. It provides a platform for recruiting multiple proteins to the DNA and thus coordinates various processes associated with replication and repair [117]. Subsets of these interactions are regulated through the modification of PCNA by ubiquitin and SUMO. PCNA is modified by ubiquitin in response to DNA-damaging agents, including MMS, 4-Nitroquinoline 1-oxide and UV light [118,119]. Such DNA damage leads to monoubiquitylation of PCNA on K164, which strengthens the interaction between PCNA and specialized ubiquitin-binding motif containing DNA polymerases, and enables synthesis through the damaged site [120]. This pathway, known as translesion synthesis, constitutes an error-prone branch of the post-replication repair (PRR) pathway (reviewed in [121]). Moreover, ubiquitin attached to K164 of PCNA can be further modified by K63 linkages to form a polyubiquitin chain [118,122]. Polyubiquitylated PCNA is a prerequisite for proceeding along the error-free branch of PRR, possibly using the template switch/gap repair mechanism [123,124].

PCNA can, however, be ubiquitylated on K164 in an alternative pathway dependent on Asf1, which is involved in the deposition of histones H3 and H4 onto newly synthesized DNA and was implicated in the processing of stalled replication forks [125]. Furthermore, persistent nicks in DNA ligase I deficient cells result in PCNA’s ubiquitylation at lysine K107 and consequent S-phase checkpoint response [126]. These observations suggest that different types of DNA damage may lead to different PCNA ubiquitylation patterns and different cellular outcomes. 

Prior to the S phase, PCNA is also modified by SUMO on lysine K164 and, to a lesser extent, on K127 [118]. While PCNA is sumoylated even in the absence of exogenous DNA damage, massive DNA damage leads to its heightened sumoylation [118]. Sumoylated PCNA (SUMO-PCNA) then recruits Srs2, which has been shown to inhibit unwanted HR and to channel DNA lesions into the PRR pathway [127,128]. SUMO-PCNA interacts with Srs2 through two interaction sites. One site is represented by SUMO interaction motif (SIM) composed of last 5 C-terminal amino acids (IIVID). The second site includes PCNA-specific interaction motif (PIM). Importantly, both sites are necessary for efficient function *in vivo* [53,129,130,131]. How exactly Srs2 inhibits HR at the replication fork is not quite clear, but two possible mechanisms have been suggested. First, the inhibitory effect may be mediated by Srs2’s ability to disrupt Rad51 presynaptic filaments [51,52]. Second, Srs2 may block the extension of recombination intermediates by outcompeting SUMO-PCNA from its complex with Polδ during the repair synthesis [132]. 

Though sumoylation and ubiquitylation target the same lysine residue of PCNA (K164) they do not compete for the substrate and rather act in concert to favor PRR [123,127]. The presumed SUMO–ubiquitin cooperation is further evidenced by the observation that both modifications of K164 are important for the break-induced repair [133]. The K164 modifications seem not to compete with PCNA-interacting proteins, since K164 is located at the back of the PCNA ring while most proteins interact at the front of that ring [134,135]. On the other hand, K127 lies at the site responsible for these interactions and it has been proposed that its sumoylation blocks interactions with most PCNA-interacting proteins [136]. The ubiquitylation and sumoylation of PCNA on K164 are conserved among eukaryotic species, and their effects share certain similarities [118,120,137,138,139,140,141]. Though PCNA sumoylation was originally thought not to be present in human cells, a recent study opposes this supposition and suggests that SUMO-PCNA recruits the PARI protein by a similar mechanism and with an effect similar to that of Srs2 [140]. 

In addition to sumoylation and ubiquitylation, mammalian PCNA has been shown to undergo phosphorylation and acetylation. Phosphorylation can stabilize PCNA and stimulate cell proliferation [142]. The different acetylation statuses lead to the appearance of three PCNA isoforms that differ in their localization and affinities towards DNA polymerases β and δ or the MTH2 protein [143,144].

## 6. Conclusions

**Figure 4:**
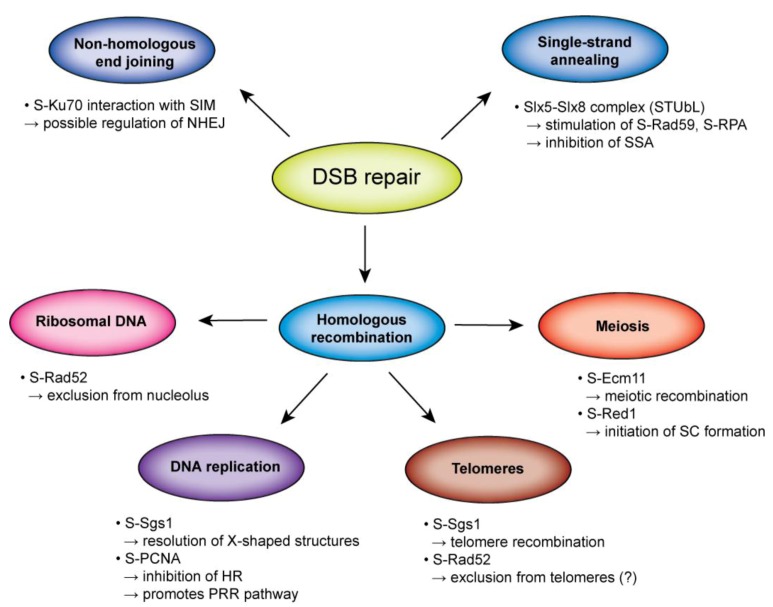
Role of sumoylation on the relationship of homologous recombination and other DNA metabolic processes in *S. cerevisiae*. Sumoylation influences not only repair of DSBs but also homeostasis of rDNA and telomeres, DNA replication, and meiosis. Examples of sumoylation’s involvement in this interplay are illustrated.

Homologous recombination is a complex multistep pathway that allows various outcomes, depending on the specific situation and subcellular localization. Moreover, HR is interlinked with various other pathways, which is underlined by the fact that they share some of the same protein factors. In such cases, regulation is paramount, and evidence suggests that sumoylation may be an important process to regulate and coordinate the interplay between HR and other pathways (Figure 4).

In some studies, sumoylation has been found to repress recombination [34,67,127,128]; in others, sumoylation has been seen to promote it [38]. It is therefore possible that sumoylation influences the utilization of positive and the suppression of negative recombination outcomes. On the molecular level, sumoylation often stimulates protein–protein interactions. Therefore, it may bring together proteins to facilitate a certain DNA repair pathway, and by doing so to block alternative repair pathways. Although the evidence supporting this idea can be found in PML bodies or PCNA-Srs2 complex formation [95,127,128], the identification of SUMO-dependent DNA repair complexes remains a challenge for future years.

Alternatively, sumoylation can also serve to disassemble or dissociate proteins after fulfilling corresponding task to allow subsequent steps and completion of HR. Future mechanistic work to understand how SUMO uses these different ways to regulate each step of HR will bring clarity and generate a more comprehensive view of the role of sumoylation. In addition, it will be interesting to understand how these regulations occur in response to DNA damage. Last but not least, understanding the role of sumoylation and its regulatory function could potentially be used in development of novel chemotherapeutic treatments.
